# The mediating role of personal values between COVID-19-related posttraumatic growth and life satisfaction among Chinese college students: A two-wave longitudinal study

**DOI:** 10.3389/fpsyg.2022.926375

**Published:** 2022-09-23

**Authors:** Jia-Qiong Xie, Hua Zhang, Xiang Zhang, Ming-Ze Yin, Jing Yang, Ke Chen, Jian-Ru Xiong, Yi-Qiang Chen

**Affiliations:** ^1^Faculty of Social Sciences, Chongqing University, Chongqing, China; ^2^Faculty of Psychology, Southwest University, Chongqing, China; ^3^Faculty of Education, Southwest University, Chongqing, China; ^4^Office of Social Sciences, Chongqing University, Chongqing, China; ^5^Department of Student Affairs, Chongqing University, Chongqing, China; ^6^School of Foreign Languages and Cultures, Chongqing University, Chongqing, China

**Keywords:** COVID-19, posttraumatic growth, life satisfaction, personal value, mediating role

## Abstract

Despite considerable disruption of social order caused by the COVID-19 pandemic, it has also been said to contribute to positive psychological changes and influence on the perception of public life satisfaction. The present study aimed to explore the association between the COVID-19 related posttraumatic growth and life satisfaction and the mediating role of personal values. A two-wave longitudinal design was used. 226 self-quarantined Chinese college students (58.8% male) completed post traumatic growth inventory (Time 1), satisfaction with life scale (Time 2), personal values questionnaire (Time 2) between February 2020 and May 2021. Results showed that more than half of self-quarantined Chinese college students reported moderate to high levels of the COVID-19 related posttraumatic growth. A structural equation model revealed that COVID-19 related posttraumatic growth was positively associated to life satisfaction, and self-transcendence and self-enhancement values partially mediated this association. These findings shed light on whether and how pandemic-related posttraumatic growth influenced personal life satisfaction, supporting the outcome and process perspectives of posttraumatic growth as well as Schwartz’s value theory. Based on the findings, some positive psychology interventions, such as online rumination activities and mindfulness practice, were proposed to enhance self-quarantined college students’ posttraumatic growth and life satisfaction.

## Introduction

The outbreak of the novel coronavirus disease in 2019 (COVID-19) caused a major public health crisis worldwide and has inflicted over 400 million infections and 6 million fatalities (WHO, 2022). Havoc on social stability and economic prosperity made this epidemic an ongoing, chronic and collective traumatic event (Cheng and Liu, 2022) with a significant decrease in life satisfaction among the public (Brooks et al., 2020). As a consequence, finding effective ways to maintain or develop public LS is a crucial issue, especially in the post-epidemic era. As famously said by Nietzsche (1998), “That which does not kill me makes me stronger,” posttraumatic growth (PTG) refers to the positive psychological changes catalyzed by struggling with trauma or extremely challenging circumstances (Tedeschi and Calhoun, 1996; Tedeschi et al., 2018). Empirically, the positive effects of PTG in enhancing LS and mental health have been extensively documented in the literature (e.g., Triplett et al., 2012; Rzeszutek et al., 2019; Mostarac and Brajković, 2022).

Despite a growing interest in the COVID-19 related PTG, current literature lacks a theoretical framework and empirical evidence that explains whether and how the COVID-19 related PTG is beneficial to the public LS. Methodologically, previous studies mostly used cross-sectional designs to assess the beneficial effects of PTG on LS. Longitudinal designs are also needed to strengthen conclusions about the temporal ordering of effects. Additionally, most empirical studies on COVID-19 related PTG mainly focus on populations directly exposed to the pandemic, such as health care workers. Some gaps, nevertheless, remain among self-quarantined college students in this line of research. In fact, stressful experiences such as the threat of death and infection, academic pressure, and poor interpersonal relationships can all contribute to low LS (Gundogan, 2021) while simultaneously creating possibilities for PTG among self-quarantined college students.

To narrow these research gaps, this study aims to investigate whether and how the COVID-19 related PTG affects LS among self-quarantined college students from a positive psychology perspective, theoretically drawing on the comprehensive model of PTG and Schwartz’s value theory, and methodologically using a two-wave longitudinal design. As a broad, ideal, cross-situational goal (Rokeach, 1973; Schwartz, 1992), personal values were introduced to explore the influence mechanism of PTG on LS, considering the shifting of personal values in the PTG process (Weiss, 2014) and the prominent role of personal values in life goal setting and cognitive evaluation (Bardi et al., 2014; Sortheix et al., 2019).

### Posttraumatic growth

Tedeschi first proposed the term “PTG” to describe positive psychological changes resulting from struggles with a highly challenging living environment or traumatic occurrences (Tedeschi and Calhoun, 1996). These positive changes involved relating to others, recognition of new possibilities, a feeling of personal strength, spiritual change, a greater appreciation of life, realignment of life priorities, and identification of new values (Tedeschi and Calhoun, 1996, 2004). The COVID-19 pandemic, as a biological disaster, is undeniably a traumatic stressor for individuals who are directly experiencing symptoms, witnessing the suffering of the patients, experiencing realistic or unrealistic fear of infection, social isolation, financial hardships, or the community at large (Chen et al., 2021). People exposed to COVID-19 may also experience PTG. Empirically, researchers have examined the prevalence of COVID-19 related PTG and found moderate to high levels detected among healthcare workers (Chen et al., 2021; Finstad et al., 2021; Lyu et al., 2021; Moreno-Jiménez et al., 2021; Yıldız, 2021; Feingold et al., 2022) and American veterans (Pietrzak et al., 2021) as well as low levels among adolescents (Jian et al., 2022; Ulset and Soest Von, 2022) and young adults (Hyun et al., 2021).

For self-quarantined college students, the challenges of moving learning sessions from face-to-face arrangement to online sessions, and the disruption of graduation and further education plans have greatly increased the academic and employment pressure (Lederer et al., 2021). Meanwhile, young college students maintain a higher frequency of inter-individual contacts, and social restriction measures would aggravate poor interpersonal relationships due to travel bans and self-isolation, among others (Birmingham et al., 2021). Furthermore, college students today are more susceptible to media sensationalism as frequent users of the internet. They are in many cases paying too much attention to negative emotions such as fear and anger, and witnessing death and suffering of ordinary people in unfortunate circumstances (Bian et al., 2020; Yuan et al., 2021). All these are risk factors for low LS (Gundogan, 2021) and severe psychological problems (Duan et al., 2020) among college students in self-quarantine beyond the threat of death and infection. However, given that challenges and suffering also provide opportunities for PTG realization, they are by far a perfect fit for COVID-19 related PTG (Chi et al., 2020) study with direct experience of the pandemic.

### Posttraumatic growth and life satisfaction

As a cognitive component of subjective well-being, LS refers to a cognitive assessment of individual’s life according to subjectively shaped standards about appropriate circumstances (Diener et al., 1985). The individual’s assessment of satisfaction is high if the subjectively perceived life circumstances match a unique set of criteria (Pavot and Diener, 1993; Mostarac and Brajković, 2022). While negative situations such as trauma, stress, and fear that the individual has experienced decrease LS, many situations such as the individual’s positive life experiences, social support, and being psychologically strong are factors that would increase LS (Gundogan, 2021).

Previous studies on other traumatic events have demonstrated that PTG can directly or indirectly improve individual LS. In meta-analysis reviews, PTG was found to be positively related to LS, an indicator of well-being (Helgeson et al., 2006), and this positive link may become stronger the more time has elapsed after the traumatic event (Park, 2004). Recently, cross-sectional studies found a positive association between PTG and LS in patients with cancer (Mostarac and Brajković, 2022) and HIV (Rzeszutek et al., 2019) and nurses exposed to work violence (Itzhaki et al., 2015). Furthermore, albeit less often, researchers have found mediators of the association between PTG and LS, such as meaning in life (Triplett et al., 2012; Mostarac and Brajković, 2022).

Theoretically, the explanations of the relations between PTG and LS have largely grouped two perspectives. From an outcome perspective, the development of PTG is theorized as positive changes in multiple dimensions of life, such as a greater appreciation of life, more intimate social relationships, heightened feelings of personal strength (Tedeschi and Calhoun, 2004), as well as the development of life narratives and wisdom (Pals and McAdams, 2004). These positive shifts in life can easily be linked to higher LS (Jayawickreme and Blackie, 2014). Some scholars even believed that the two constructs of PTG and subjective well-being are essentially similar (Joseph and Linley, 2005). From a process perspective, the comprehensive model of PTG proposed that a traumatic event shakes or destroys core beliefs and then triggers rumination progresses to rebuild or repair it (Tedeschi and Calhoun, 2004). Rumination progresses from initially automatic (e.g., intrusive memories and images) to more deliberate (e.g., analyzing the new situation and re-appraisal; Zoellner and Maercker, 2006). Such constructive cognitive activities directly reduce emotional distress, promote individuals’ ability to adapt to the traumatic environment and maintain or improve LS (Calhoun et al., 2010; Calhoun and Tedeschi, 2014). In addition, PTG may indirectly influence LS through reconstructed core beliefs such as clarity of life priorities (Joseph and Linley, 2005). We explore this in detail below. In sum, PTG is seen as both a process and an outcome—it is a positive outcome in and of itself, but the process of coming to terms with trauma and identifying positive changes is a long-term process that may also result in greater LS in the long run (Jayawickreme and Blackie, 2014).

### Mediating roles of personal values

To further explore the underlying psychological mechanism that explains the positive effect of the COVID-19 related PTG on LS, we resort to personal values. According to the comprehensive model of PTG, the process of PTG involves core belief reconstruction such as value clarification and changing priorities (Tedeschi and Calhoun, 2004; Weiss, 2014). These individuals’ value priorities shifting may promote cognitive changes in perception of traumatic events (Vecchione et al., 2012), retrieval of traumatic memories (Xiu et al., 2017), and choice of coping strategies (Tweed and Conway, 2006), affecting people’s evaluation of life. Therefore, PTG may have a positive indirect effect on LS by influencing personal values.

The dominant model in the personal value literature is the one proposed by Schwartz (1992). Regarding personal value content, this theory has evolved from the original 10 basic personal values (Schwartz, 1992) to the refined 19 (Schwartz et al., 2012). These values form a circle structure (see Schwartz et al., 2012, p. 669) based on the degree of compatibility or conflict between the goals expressed. The array of values represents a motivational continuum, i.e., values placed next to each other have more compatible motivations (e.g., benevolence and universalism), whereas those placed further apart are more conflicting (e.g., power and universalism). Schwartz (1992) grouped these values into two pairs of higher-order value dimensions: openness to change versus conservation, and self-enhancement (SE) versus self-transcendence (ST). Additionally, the circle of values is a continuum, in which values blend into one another rather than forming discrete entities (Schwartz, 1992; Schwartz et al., 2012). This implies that one can partition the value circle arbitrarily into as many or as few value categories as is convenient (Sagiv and Schwartz, 2022).

In this study, SE (expressed by power, achievement, and face values) and ST (expressed by humility, benevolence, and universalism values) values were mainly considered to analyze the influence mechanism of the COVID-19 related PTG on LS. The reason is that compared with other traumatic events, exposure to collective traumatic events (e.g., earthquakes and the COVID-19 epidemic) shifts people’s values toward prosocial directions (Oishi et al., 2017), and the PTG of collective traumatic events involves not only positive changes in individual life but also group-communal strength and social benefits, especially in collectivist cultures such as China (Wlodarczyk et al., 2017). A national representative study has also found that the COVID-19 related PTG is associated with high identification with all humanity and priority given to human rights over self-interest (Vazquez et al., 2021). Therefore, the SE and ST values capture the conflict between concern for the welfare and interests of others, and concern for one’s material possessions, relative success (Sagiv and Schwartz, 2022), are more appropriate than other value categories to be considered in COVID-19 related PTG research.

The indirect effect of PTG on LS may be achieved by raising the ST value priority and lowering the SE value priority. Reprioritizing personal values was a crucial theme of PTG. For individuals experiencing PTG, new priorities included a greater appreciation of one’s own internal self-worth rather than material possessions and a new understanding of other people, which led to greater compassion and the drive towards altruism (Martin et al., 2017). For example, burn-related PTG studies found that burn survivors had a greater appreciation for their health and well-being, as well as for family and friends (McLean et al., 2015; Martin et al., 2016). Evidence from collective traumatic events studies also reported a positive association between PTG and prosocial behaviour and altruism among refugees exposed to violence (Canevello et al., 2021) and adolescents who experienced earthquake (Liu et al., 2021). It was clear that the reminder of one’s mortality drove appreciation of the present moment, with regard to both one’s own personal and spiritual growth as well as the wellbeing of others.

Personal values, as a cognitive representation of goals to be pursued (Sagiv and Schwartz, 2022), affect the subjectively shaped standards of appropriate circumstances based on which individuals evaluate life quality. Thus, individuals with different value orientations have different subjective LS. One theoretical perspective that proposes an effect of personal values on LS is the “healthy values” perspective (Sagiv and Schwartz, 2000; Sortheix and Schwartz, 2017). This perspective argues that pursuing growth values (e.g., benevolence and universalism) enhances well-being because these values are self-actualizing. In contrast, pursuing deficiency values (e.g., face and power) is believed to lead to perceptions, attitudes, or behaviors that reduce well-being. Schwartz et al. (2012) suggested that the pursuit of ST is relatively anxiety-free and fosters self-expansion and growth. It is considered a healthy growth value that can promote individual LS (Sortheix and Lönnqvist, 2014; Sagiv and Schwartz, 2022). In contrast, SE value is seen as an unhealthy deficiency or self-protective value that reflects the pursuit of avoiding anxiety and self-preservation, leading to lower satisfaction (Dittmar et al., 2014; Sagiv and Schwartz, 2022). Thus, ST will be associated with more, and SE with less, LS.

**Figure 1:**
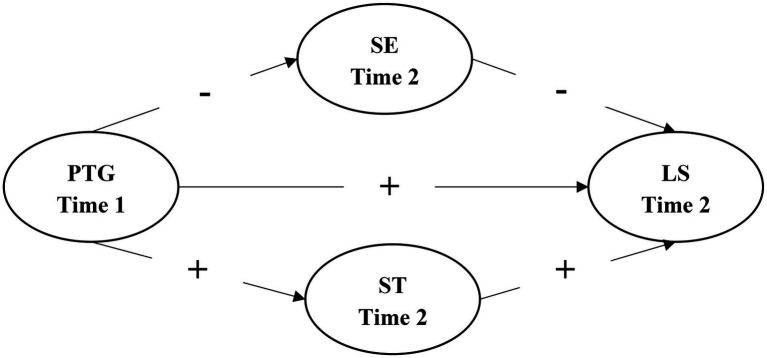
Hypothesized structural equation model of the relationships between the COVID-19 related posttraumatic growth (PTG), life satisfaction (LS), self-enhancement value (SE), and self-transcendence value (ST). Time 1 = the peak phase of the COVID-19 pandemic in China, Time 2 = the post-pandemic phase of the COVID-19 in China.

In sum, the COVID-19 pandemic was a traumatic event for self-quarantined college students while also providing them with an opportunity for PTG realization. In the long run, COVID-19 related PTG may have a direct effect on their LS, as well as an indirect effect mediated by personal values. Drawing on the comprehensive model of PTG and Schwartz’s personal value theory, we proposed a research model shown in Figure 1 to examine the long-term positive effect of COVID-19 related PTG on LS and the mediating role of ST and SE values among self-quarantined college students. To this end, a two-wave longitudinal design was conducted to look into the following hypotheses:

*Hypothesis 1*: The COVID-19 related PTG positively predicts LS.

*Hypothesis 2*: The COVID-19 related PTG negatively predicts SE value (H2-a) and positively predicts ST value (H2-b).

*Hypothesis 3*: SE value negatively predicts LS (H3-a) and ST value positively predicts LS (H3-b).

*Hypothesis 4*: The correlation between PTG and LS is mediated by SE (H4-a) and ST values (H4-b).

## Materials and methods

### Participants and procedures

Data were collected from February 27, 2020 (the peak phase of the COVID-19 in China, all universities in China have suspended offline classes and students have been asked to stay confined at home, Time 1) with a 1-week duration until May 5, 2021 (the post-pandemic phase in China, Time 2), with a 2-week duration. At Time 1, 300 self-quarantined undergraduate students from two universities in southwest China were recruited. Participants come from different provinces all over the country. 33 participants who had self-quarantined at home for less than 1 month were excluded, and 267 valid questionnaires were retained. One year later (Time 2), 41 withdrew from the study, and 226 adults from Time 1 were retained. Therefore, the final sample consisted of 226 participants between 18 and 23 (M_age_ = 19.64, SD_age_ = 0.87, Male 58.8%) who completed the two-wave survey. G*Power was employed to calculate the sample size required for this study (Erdfelder et al., 2009). *A priori* analysis showed that a sample size of 187 was required to detect a small effect size of 0.1 with a power of 0.95 (3 predictors). Thus, 226 subjects were an adequate sample size.

Both surveys were performed online using *SOJUMP*,^1^ a popular online survey platform in China, after being announced on students’ social media groups. Participants voluntarily clicked the link in the announcement to access the survey platform after learning about the objectives and design of the study. They then completed the questionnaire after providing an informed consent. Participants in the initial survey were encouraged to take part in a follow-up survey 1 year later. If they consented, participants were requested to supply valid contact information (e.g., email addresses). To control the common method bias, procedurally, we ensured the anonymity of all participants and reminded them to answer according to the actual situation. Additionally, acquiescence was reduced by reverse scoring some of the items. The Chongqing Ninth People’s Hospital Review Board, China, approved this study (approval number: 2019016).

### Measures

#### Posttraumatic growth

The Post Traumatic Growth Inventory (PTGI) developed by Tedeschi and Calhoun (1996) was adapted to measure the COVID-19 related PTG at Time 1. This inventory is composed of 21 items grouped into five subscales: relating to others (seven items), new possibilities (five items), personal strength (four items), spiritual change (two items), and appreciation of life (three items). Instructions of the PTGI were modified to index experiences associated with the COVID-19 pandemic: e.g., “Please indicate the degree to which you experienced these changes in your life as a result of the COVID-19 pandemic: knowing I can handle difficulties.” The responses were based on a six-point Likert scale, where a score of zero indicates ‘not at all’ and a score of five indicates ‘very great’. Higher scores indicate more growth after the experience of the COVID-19 pandemic. The overall Cronbach’s α for PTGI was 0.95. The coefficients for each of its domains were: relating to others (0.87), new possibilities (0.82), personal strength (0.79), spiritual change (0.71), and appreciation of life (0.73).

#### Life satisfaction

Satisfaction with Life Scale (SWLS) developed by Diener et al. (1985) was used to evaluate individual’s overall LS at Time 2. The questionnaire includes five statements on a five-point Likert scale, where a score of one indicates ‘strongly disagree’ and a score of seven indicates ‘strongly agree’. Higher scores indicate more LS. A representative item was “In most ways, my life is close to my ideal.” The scale showed high internal consistency (Cronbach’s α = 0.86) in the current study.

#### Personal values

Personal values at Time 2 were assessed by the Personal Values Questionnaire (PVQ) developed by Schwartz et al. (2012). The current study focuses on SE and ST value dimensions in Schwartz’s value theory, which are measured by 25 items. ST value dimension consists of benevolence (5 items), universalism (8 items), and humility values (2 items). SE value dimension consists of achievement (3 items), power (5 items), and face (2 items) values. Each item offers verbal portraits of different people, and respondents were asked to evaluate how similar they are to the portrait. A sample item was “he/she wants people to do what he/she says.” Items were scored on a six-point scale, where a score of one indicates ‘not like me at all’ and a score of six indicates ‘very much like me’. In this sample, the Cronbach’s α of SE and ST value dimensions were 0.85 and 0.97 respectively, indicating high internal consistency.

### Analytical strategy

First, a series of preliminary analyses were conducted: (1) common method bias analysis using the Harman one factor test, (2) calculating the mean (M) and standard deviations (SD) of the study variables, and (3) testing the gender and age differences in the study variables, and (4) calculating the correlation between the study variables. The SPSS 22.0 statistical programme (SPSS, Chicago IL, United States) was used in preliminary analyses. All *p* values were two-sided, and those less than 0.05 were considered statistically significant.

Next, we tested the measurement model by calculating the convergent validity and discriminant validity to ensure a satisfactory model to proceed to the path analysis. The following criteria were adopted: (1) factor loadings higher than the minimum threshold of 0.4 (Tabachnick and Fidell, 2007), (2) composite reliability (CR) higher than the recommended threshold of 0.70 (Gefen et al., 2000), (3) average variance extracted (AVE) higher than 0.5 (Sarstedt et al., 2022), and (4) the square root of AVE for each construct greater than its correlations with other constructs (Sarstedt et al., 2022).

Then, structural equation modeling (SEM) was employed to estimate the mediation effects with Amos 22.0 (Arbuckle, 2013). The default estimation of the maximum likelihood method was used. To study the adequacy of the estimated model, we determined good model fit if the *χ^2^*/*df* < 3 and the Comparative Fit Index (CFI) and Tucker-Lewis Index (TLI) were greater than 0.9 as recommended by Salisbury et al. (2002). The Root Mean Square Error of Approximation (RMSEA) and Standardized Root Mean Square Residual (SRMR) values less than or equal to 0.08 represent an acceptable fit (Bryne, 2013).

Finally, the Bootstrap method (5,000 samples) was used to evaluate the significance of the mediating effect. Bootstrapping generated a large sample from the data set and estimated the bias-corrected 95% confidence intervals (CI) for the indirect effect, indicating a significant indirect effect when the CI did not include 0 (Cheung and Lau, 2008).

## Results

### Preliminary analyses

The Harman single factor test has been conducted to ensure that the dataset is free of common technique bias. The statistical results found that the highest covariance explained by one factor is 27.15%, lower than the cut-off value of 50% (Harman, 1967), indicating that none of the factors significantly dominated the explanation of the variance. Thus, the common method bias was not a serious concern in this study.

Table 1 presents the means and standard deviations of all study variables. The results indicated a moderate level of PTG and LS, as well as high level of ST and SE values among self-quarantined Chinese college students. Regarding the specific dimensions of PTG, spiritual change scored the highest, followed by appreciation of life, personal strength, relating to others, and new possibilities. Given that the total and subscale scores were non-normally distributed (all Shapiro–Wilk test *p* values <0.0001), scores were dichotomized so that responses of “moderate,” “great,” or “very great” growth indicated a positive endorsement of PTG (Pietrzak et al., 2021; Feingold et al., 2022). In the current sample, 65.4% endorsed at least moderate levels of PTG. Regarding the specific dimensions of PTG, the most prevalent domains are spiritual change (69.5%), followed by appreciation of life (64.2%), personal strength (60.2%), relating to others (56.6%), and new possibilities (45.1%).

**Table 1:** Descriptive statistics, convergent validity, and construct reliability results.

Constructs	Indicators	M	SD	Loading	CR	AVE
PTG		3.32	0.98		0.93	0.73
	Relating to others	3.25	1.09	0.88		
	New possibilities	2.97	1.10	0.90		
	Personal strength	3.37	1.14	0.89		
	Spiritual change	3.56	1.27	0.81		
	Appreciation of life	3.45	0.99	0.78		
LS		3.32	0.75		0.86	0.57
	LS1	3.54	0.87	0.84		
	LS2	3.50	0.87	0.75		
	LS3	3.72	0.89	0.89		
	LS4	3.23	0.95	0.68		
	LS5	3.00	1.04	0.55		
SE		4.00	0.79		0.75	0.51
	Power	4.23	0.91	0.68		
	Achievement	3.71	1.01	0.69		
	Face	4.26	0.93	0.75		
ST		4.99	0.86		0.90	0.76
	Humility	4.70	1.06	0.73		
	Benevolence	5.05	0.90	0.94		
	Universalism	5.05	0.89	0.93		

All loadings significant at the *p* < 0.001 level; LS1-LS5 = 5 items of life satisfaction.

Independent sample *t*-test results show that there were no gender and age differences in all study variables. Correlation analysis shows that PTG was positively correlated with ST value and LS, PTG was negatively correlated with SE value; ST value was positively correlated with LS, and SE value was negatively correlated with LS (see Table 2).

**Table 2:** Inter-construct correlation matrix.

	PTG	SE	ST	LS
PTG	(0.85)			
SE	−0.24^**^	(0.75)		
ST	0.16^*^	−0.40^**^	(0.71)	
LS	0.53^**^	−0.23^**^	0.30^**^	(0.87)

Square roots of average variances extracted are shown in parentheses on the diagonal.

* *p* < 0.05 and

** *p* < 0.01.

### Testing the measurement model

The factor loadings, CR, and AVE values were evaluated to confirm convergent validity. The results revealed factor loadings, CR, and AVE values (see Table 1) to be greater than the threshold value mentioned above, demonstrating acceptable convergent validity. The discriminant validity of the constructs was also assessed by contrasting the square root of the AVE values for each construct with the inter-construct correlations. The square roots of the AVE values (presented in parentheses in Table 2) were larger than the respective inter-construct correlations, indicating acceptable discriminant validity.

### Testing the structural model

**Figure 2:**
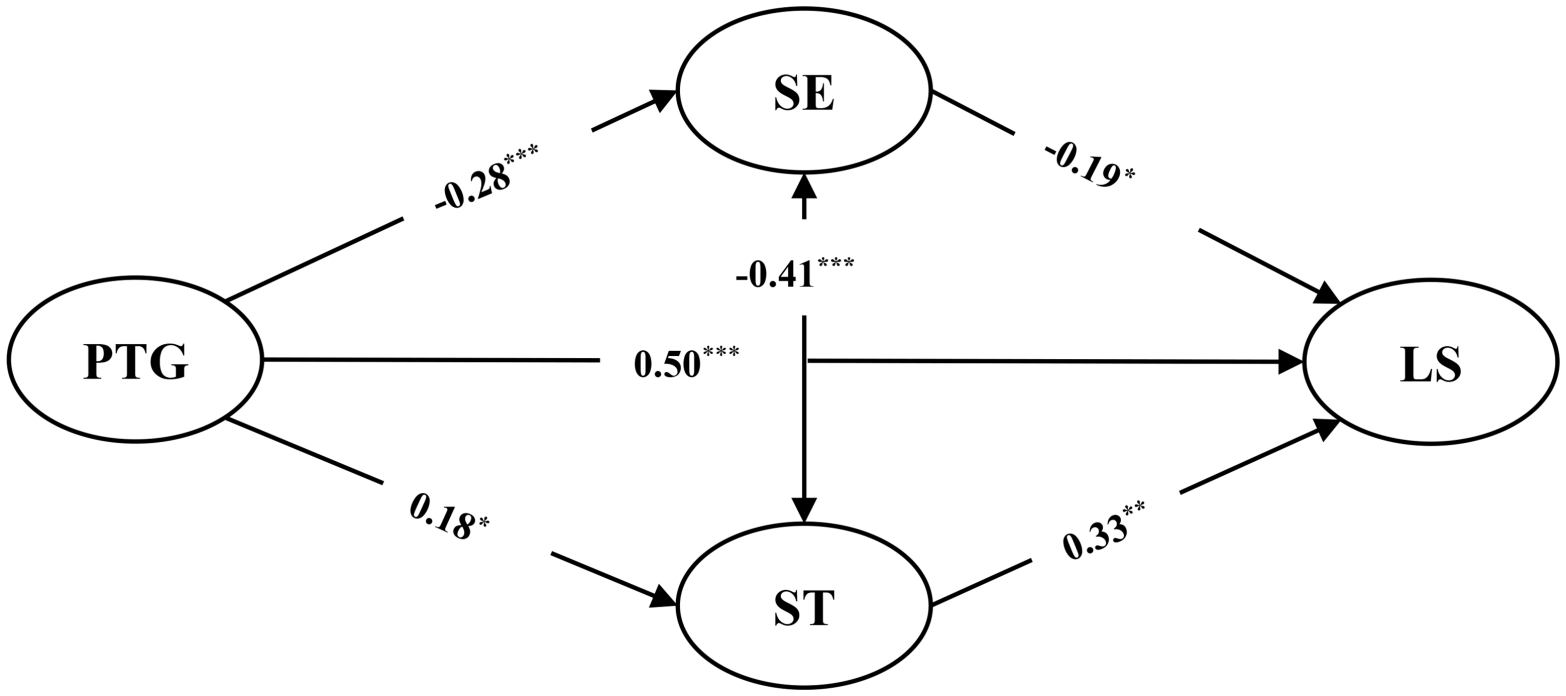
The mediation model of SE and ST values between COVID-19 related PTG and LS. Path coefficients are standardized. ^*^p < 0.05, ^**^p < 0.01, ^***^p < 0.001.

With PTG (Time 1) as the independent variable, SE and ST values (Time 2) as the mediating variables, and LS (Time 2) as the dependent variable, the mediating effect model was constructed (see Figure 2). Previous studies have shown that there is a relatively robust correlation between SE and ST values (Schwartz et al., 2012), and a moderate correlation is also detected in the above correlation analysis. Therefore, a correlation path is established between SE and ST values. This model showed a good fit to the data [*χ^2^*/*df* = 1.81, *p* > 0.05, CFI = 0.96, TLI = 0.96, RMSEA = 0.06 (90% CI for RMSEA = 0.05, 0.07), SRMR = 0.05].

The results revealed that PTG was found to be a positive and significant predictor of LS (*β* = 0.50, *p* < 0.001) and ST value (*β* = 0.18, *p* < 0.05), but a negative and significant predictor of SE value (= −0.28, p 0.001). Thus, Hypotheses 1 and 2 are supported. It was also found that SE value is a negative and significant predictor of LS (*β* = −0.19, *p* < 0.05), while ST value is a positive and significant predictor of LS (*β* = 0.33, *p* < 0.001), supporting Hypothesis 3.

### Testing the meditation effects

We conducted a bootstrapping analysis (5,000 samples) to test the mediating effects of personal values. The results showed that the mediating effects of SE and ST values on the association between PTG and LS were 0.05 and 0.06, respectively, and the 95% confidence intervals for both mediating effects do not include 0. Furthermore, the total effect of PTG on LS was 0.61, among which, the total indirect effect of PTG on LS through SE and ST values was 0.11, accounting for 18.33% of the total effect. The coefficients and confidence intervals of direct and indirect effects are presented in Table 3. These results indicate that the partial mediation of SE and ST values between PTG and LS were observed, as in Hypothesis 4.

**Table 3:** Testing the mediation effects of SE and ST values between PTG and LS.

**Paths**	***β***	**SE**	**95% CI**
**Direct effect**			
PTG → LS	0.50	0.08	[0.34, 0.66]
**Indirect effect**			
Total indirect effect	0.11	0.04	[0.08, 0.14]
PTG → SE → LS	0.05	0.02	[0.04, 0.07]
PTG → ST → LS	0.06	0.02	[0.04, 0.07]
**Total effect**			
PTG → LS	0.61	0.05	[0.50, 0.70]

The effect coefficients are standardized.

## Discussion

This study assessed the positive impact of the COVID-19 pandemic on individuals’ perception of life from the perspective of positive psychology. Using a longitudinal design with data collected from two waves, our results demonstrated that the COVID-19 related PTG was a positive factor of self-quarantined college students’ LS 1 year after the outbreak, and personal values played a mediating role in this relationship. These findings enrich research on public well-being under the COVID-19 pandemic and help illuminate how coronavirus-related PTG contributes to personal LS.

### COVID-19 related posttraumatic growth and life satisfaction

The results showed that 65.4% of participants experienced at least medium levels of positive changes as a result of the COVID-19 pandemic, most commonly greater spiritual change and appreciation of life. These results were supported by a previous study, which found that 66.9% of Chinese college students had experienced PTG during the COVID-19 pandemic (Chi et al., 2020). Additionally, while the prevalence of the COVID-19 related PTG in the present sample is lower than the 77% rate reported among healthcare workers (Feingold et al., 2022), it is markedly higher than the previous prevalence observed among home-confined Chinese adolescents (20.6%; Jian et al., 2022), Norwegian adolescents (9.6%; Ulset and Soest Von, 2022), and American veterans (43.3%; Pietrzak et al., 2021). Different cut-off values, the time after the disaster, cultural context, or age disparities might all have contributed to these discrepancies. Our findings shed light on a reassuring fact that if self-quarantined college students without direct exposure to COVID-19 can see this crisis as a positive turning point, engage in deeper rumination and insight into their own life, they can also experience similar PTG to those who have been directly exposed to COVID-19, such as healthcare workers.

The current study found that PTG at the peak phase of the COVID-19 pandemic was positively associated subsequent LS 1 year later. Although the long-term effect of COVID-19 related PTG on LS has seldom been examined, our results are comparable to previous cross-sectional studies, which reported a positive relationship between PTG and LS observed among cancer survivors (Mostarac and Brajković, 2022), people with physical disabilities (Kim et al., 2016), as well as myocardial infarction survivors (Ogińska-Bulik, 2014). This finding suggested that COVID-19 related PTG is also equally beneficial to individuals’ LS in the long run, extending the positive effect of PTG on LS to pandemic diseases. This direct effect of PTG on LS reinforced the outcome perspective of PTG, which held that PTG as a positive change in individuals’ life after struggling with trauma involved awareness of their previously undiscovered strengths and appreciation of life, among other things (Janoff-Bulman, 2004).

### The moderating role of personal values

The effect mechanism of PTG on LS is explained for the first time in this work using personal values. Given the collective traumatic nature of COVID-19, we proposed that the indirect effect of PTG on LS may be achieved by raising the ST value priority and lowering the SE value priority based on Schwartz’s personal value theory. As expected, the association between COVID-19 related PTG at Time 1 and LS at Time 2 was partially mediated by ST and SE values at Time 2. This indirect effect of PTG on LS through personal values reinforced the process perspective of PTG, which held that changing values priority is an essential process for achieving PTG and will affect the life goals setting and life quality assessment in the post-traumatic surroundings and even in the future (Nolen-Hoeksema and Davis, 2004; Martin et al., 2017). Additionally, the opposite correlation direction of ST and SE values with PTG and LS also extended the conflict of goals expressed by these two values, as postulated in Schwartz’s (1992) personal value theory, to the conflict of relationship between the two values and other psychological constructs.

Each of the individual links in our mediation model is worth noting. We found that PTG at Time 1 was positively related to ST value, while negatively related to SE value at Time 2. The results are consistent with the collective trauma study found that Chileans who experienced earthquakes-related PTG, reported greater group strengths and an emphasis on social benefits (Wlodarczyk et al., 2017). This finding suggested that the COVID-19 related PTG may prompt self-quarantined college students to value others’ interests more than their own, indicating an enhanced collectivist tendency. Indeed, In deliberative rumination about the COVID-19 pandemic trauma, a core process of PTG, social supports from the country (e.g., free testing and treatment), groups, and even strangers (e.g., donations from social groups) catalyzed self-quarantined college students to value more collective interests (Sibley et al., 2020; Cheng and Liu, 2022). In the meantime, we also found that ST value was linked to higher LS, while SE value pointed to the opposite. This result is consistent with research findings that individuals with ST value rather than SE value report higher LS, especial in collectivist cultures such as China (Fischer and Boer, 2011; Du et al., 2014). This result supported the healthy values perspective, which held that pursuing values that satisfy psychological needs for growth and self-actualization promotes LS directly, whereas pursuing values that promote self-aggrandizement and self-interest undermines LS (Sagiv and Schwartz, 2000; Sortheix and Schwartz, 2017).

### Limitations

The following limitations of the current study should be addressed. First, a self-reported measure was used in this investigation. Although we adopted anonymous online survey according to Tourangeau (2014), this assessment method still cannot completely avoid the influence of response bias, such as social desirability on the results. Conceivably, individuals may overstate or understate in their responses based on social expectations, especially in personal value surveys. Future studies are suggested to use multiple approaches (e.g., observational or experimental methods) and multi-informant methods. Second, despite the longitudinal nature of the study, causal inferences should be avoided because PTG and LS were not evaluated repeatedly. Longer-term follow-up studies with repeated assessments (e.g., autoregressive cross-lagged panel designs) will be helpful in examining the causal relationship between PTG and LS. Third, in this study, based on the Schwartz’s personal values theory, only the mediating effects of ST and SE values were considered. Studies focusing on other values categories (such as work values, life values) should be encouraged. Finally, this study has a small sample size, with all participants being Chinese college students. As a result, the generalization of the findings obtained from this study is limited, even if the results are consistent with previous studies. Future studies with a larger sample size are recommended to replicate the present findings.

### Implications

There are some key theoretical and practical implications of this study. Theoretically, this study contributes to the necessary knowledge about the positive impact of COVID-19 trauma by providing a theoretical framework to explain whether and how COVID-19 related PTG could benefit LS. The direct long-term positive association between COVID-19 related PTG and LS, as well as the indirect association through raising ST value and lowering SE value revealed by this study supported the outcome and process perspectives of PTG, respectively. Simultaneously, the opposite correlation direction of ST and SE values with PTG and LS that we examined also supported the conflict of goals expressed by these two values and extended it to the relationship between two values and psychological constructs, reinforcing Schwartz’s value theory.

Practically, this research found ways to promote self-quarantined college students’ LS from the perspective of positive psychology. Based on the findings of the long-term positive association between PTG and LS, and the mediating role of personal values, psychologists should implement more positive psychology interventions to assist students in struggling with distress and amplifying positive emotions and experiences in their current life. For instance, the SEARCH framework proposed by Waters and Loton (2019) could be employed to promote PTG and LS through six pathways: strengths, emotional management, attention and awareness, relationships, coping, and habits and goals. Given the outbreak of COVID-19, some online rumination activities and mindfulness practice should be encouraged. For example, teachers can encourage students to engage in positive writing to explicitly identify positive experiences and unrecognized personal strengths that have occurred during COVID-19, to clarify their value priorities, and to identify positive possibilities in the aftermath of the pandemic (Waters et al., 2021).

## Conclusion

The current study has found that (1) more than half of self-quarantined Chinese college students reported moderate to high levels of the COVID-19 related PTG; (2) PTG during the peak phase of the COVID-19 pandemic positively predicted a year-later subsequent LS and ST value and a negative SE value; (3) ST value was positively associated with LS, while SE value presented a negative association; and (4) ST and SE values partially mediated the effect of PTG on LS.

## Data availability statement

The original contributions presented in the study are included in the article/supplementary material; further inquiries can be directed to the corresponding author.

## Ethics statement

The studies involving human participants were reviewed and approved by the Chongqing Ninth People’s Hospital Review Board, China. The patients/participants provided their informed consent to participate in this study.

## Author contributions

J-QX: conceptualization, methodology, formal analysis, investigation, writing - original draft, and editing. HZ: conceptualization, investigation, and writing – review. XZ: methodology, investigation, and writing - review. M-ZY: formal analysis and investigation. JY: formal analysis and investigation. KC: conceptualization, resources, funding acquisition, supervision, writing - review, and editing. J-RX: conceptualization, methodology, investigation, and resources. Y-QC: conceptualization, methodology, and writing - review and editing. All authors contributed to the article and approved the submitted version.

## Funding

This work was supported by the Fundamental Research Funds for the Central Universities (2022CDJSKZX07), Project of Chongqing Municipal Programs for Social Science (2021ZTZD10), Project of the Chongqing Municipal Committee of Science and Technology (cstc2021jsyj-zzysbAX0076), College Ideological and Political Work Cultivation Project of the Ministry of Education (JSZS2021-2), Project of the China Association of Higher Education (2020FDD07), Projects of the Chongqing Municipal Education Commission of China (19SKZDZX13 and 19SKSZ001), and Fundamental Research Funds for the Central Universities (2018CDJSK01XK04).

## Conflict of interest

The authors declare that the research was conducted in the absence of any commercial or financial relationships that could be construed as a potential conflict of interest.

## Publisher’s note

All claims expressed in this article are solely those of the authors and do not necessarily represent those of their affiliated organizations, or those of the publisher, the editors and the reviewers. Any product that may be evaluated in this article, or claim that may be made by its manufacturer, is not guaranteed or endorsed by the publisher.

## Footnotes

^1^ http://www.sojump.com
